# Feasibility of controlling hepatitis E in Jiangsu Province, China: a modelling study

**DOI:** 10.1186/s40249-021-00873-w

**Published:** 2021-06-29

**Authors:** Meng Yang, Xiao-Qing Cheng, Ze-Yu Zhao, Pei-Hua Li, Jia Rui, Sheng-Nan Lin, Jing-Wen Xu, Yuan-Zhao Zhu, Yao Wang, Xing-Chun Liu, Li Luo, Bin Deng, Chan Liu, Jie-Feng Huang, Tian-Long Yang, Zhuo-Yang Li, Wei-Kang Liu, Wen-Dong Liu, Ben-Hua Zhao, Yue He, Qi Yin, Si-Ying Mao, Yan-Hua Su, Xue-Feng Zhang, Tian-Mu Chen

**Affiliations:** 1 State Key Laboratory of Molecular Vaccinology and Molecular Diagnostics, School of Public Health, Xiamen University, 4221-117 South Xiang’an Road, Xiang’an District, Xiamen City, 361102 Fujian Province People’s Republic of China; 2 Jiangsu Center for Disease Control and Prevention, Nanjing City, Jiangsu Province People’s Republic of China; 3 Cirad, UMR 17, Intertryp, Université de Montpellier, 34398, Montpellier, France

**Keywords:** Hepatitis E, Transmission dynamic model, Meteorological factor, Intervention, Transmissibility

## Abstract

**Background:**

Hepatitis E, an acute zoonotic disease caused by the hepatitis E virus (HEV), has a relatively high burden in developing countries. The current research model on hepatitis E mainly uses experimental animal models (such as pigs, chickens, and rabbits) to explain the transmission of HEV. Few studies have developed a multi-host and multi-route transmission dynamic model (MHMRTDM) to explore the transmission feature of HEV. Hence, this study aimed to explore its transmission and evaluate the effectiveness of intervention using the dataset of Jiangsu Province.

**Methods:**

We developed a dataset comprising all reported HEV cases in Jiangsu Province from 2005 to 2018. The MHMRTDM was developed according to the natural history of HEV cases among humans and pigs and the multi-transmission routes such as person-to-person, pig-to-person, and environment-to-person. We estimated the key parameter of the transmission using the principle of least root mean square to fit the curve of the MHMRTDM to the reported data. We developed models with single or combined countermeasures to assess the effectiveness of interventions, which include vaccination, shortening the infectious period, and cutting transmission routes. The indicator, total attack rate (*TAR*), was adopted to assess the effectiveness.

**Results:**

From 2005 to 2018, 44 923 hepatitis E cases were reported in Jiangsu Province. The model fits the data well (*R*^2^ = 0.655, *P* < 0.001). The incidence of the disease in Jiangsu Province and its cities peaks are around March; however, transmissibility of the disease peaks in December and January. The model showed that the most effective intervention was interrupting the pig-to-person route during the incidence trough of September, thereby reducing the *TAR* by 98.11%, followed by vaccination (reducing the *TAR* by 76.25% when the vaccination coefficient is 100%) and shortening the infectious period (reducing the *TAR* by 50.05% when the infectious period is shortened to 15 days).

**Conclusions:**

HEV could be controlled by interrupting the pig-to-person route, shortening the infectious period, and vaccination. Among these interventions, the most effective was interrupting the pig-to-person route.

**Graphic Abstract:**

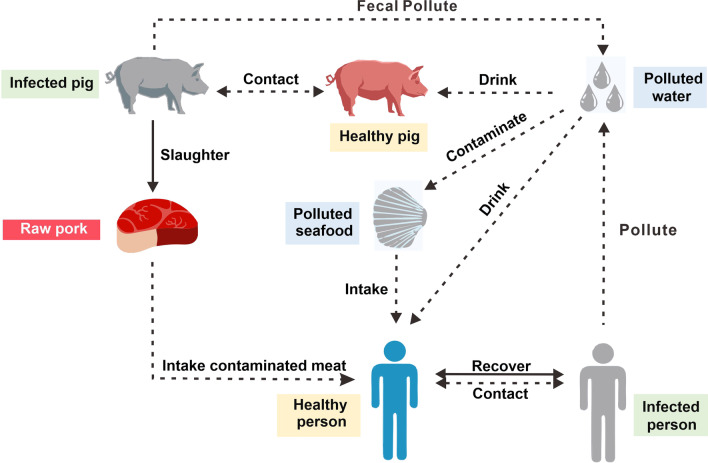

**Supplementary Information:**

The online version contains supplementary material available at 10.1186/s40249-021-00873-w.

## Background

Hepatitis E is an acute zoonotic disease caused by hepatitis E virus (HEV). The virus is mainly transmitted through the faecal-oral route upon ingestion of contaminated water and food [1]. A small percentage is transmitted through vertical and extraintestinal routes [2–4]. Among the four main genotypes of HEV, genotype 1 (HEV-1) and genotype 2 (HEV-2) are found in water and the external environment and only infect humans [5], whereas genotype 3 (HEV-3) and genotype 4 (HEV-4) can simultaneously infect humans and animals, such as pigs [6]. Consumption of undercooked pork is one of the main causes of infection, and the rate of HEV infection caused by direct contact with pigs is high [7–10]. A Swedish study on the positive rates of HEV in Sweden showed that the positive rates for HEV IgG were 13% and 9.3%, respectively, in pig farmers and the control group, respectively [11]. Currently, the major HEV genotype in China has changed from HEV-1 to HEV-4 [12]. Therefore, the main causes of infection in China are contact with infected pigs and humans, as well as environmental factors.

Globally, acute viral hepatitis has become a major public health problem and is now considered to be comparable with the three major infectious diseases (HIV/AIDS, malaria, and tuberculosis) [13]. According to the estimates of studies on the global burden of hepatitis E, approximately 20.1 million people are infected with HEV yearly and 3.4 million symptomatic cases are reported annually [14]. Many developing countries, including those in Asia (such as India, China, and Myanmar), Africa (Sudan, Somalia, Uganda), and North America (Mexico), show a high incidence of HEV infection [15, 16]. In China, the highest incidence is concentrated in the northwest and east regions, such as in Jiangsu Province [17, 18]. Therefore, the analysis of hepatitis E in Jiangsu Province is of great significance.

The current research model on hepatitis E mainly uses experimental animal models (such as pigs, chickens, and rabbits) to explain the transmission of HEV [19, 20]. Mathematical models [Discrete Poisson model, Auto Regressive Integrated Moving Average (ARIMA) model, and logistic model] can analyse the influencing factors of the disease and predict its developing trend [21–23]. A study used the susceptible-infectious-recovered (SIR) model to explain the person-to-person transmission of HEV [24]. Another study included the effect of water/food (W) to the SIR model to explore the environment-to-person route and simulate interventions for hepatitis E [25]. However, no study has developed a multi-host and multi-route transmission dynamic model (MHMRTDM) to explore the transmission features of HEV.

In this study, the MHMRTDM was used to simulate the dataset of Jiangsu Province, China. The main factors affecting the incidence and transmissibility of HEV were also investigated. Several measures were employed to evaluate the effects of interventions, including cutting off the routes of transmission, reducing the infectious period, and vaccination.

## Methods

### Research setting

Jiangsu Province is located on the eastern coast of China, with numerous lakes and complex water systems. It covers 107 200 km^2^ and administers over 13 prefecture-level cities. According to the Statistical Yearbook of Jiangsu Province, the resident population of Jiangsu Province at the end of 2019 was 80.7 million, making it the most densely populated province in China. Jiangsu Province has a transitional climate from a temperate to a subtropical zone, with mild weather, moderate rainfall, and four distinct seasons.

### Data collection

This study collected information regarding hepatitis E cases from 13 cities in Jiangsu Province from 1 January 2005 to 31 December 2018. Data were provided by the Jiangsu Provincial Center for Disease Control and Prevention (CDC) and a hepatitis E epidemic dataset, containing data on almost 45 000 cases (variables included number, gender, age, and date of onset), was established. Demographic data, including the total number of people, birth rate, and natural death rate, were obtained by consulting the Jiangsu Statistical Yearbook 2019.

### The MHMRTDM model without intervention

**Fig. 1:**
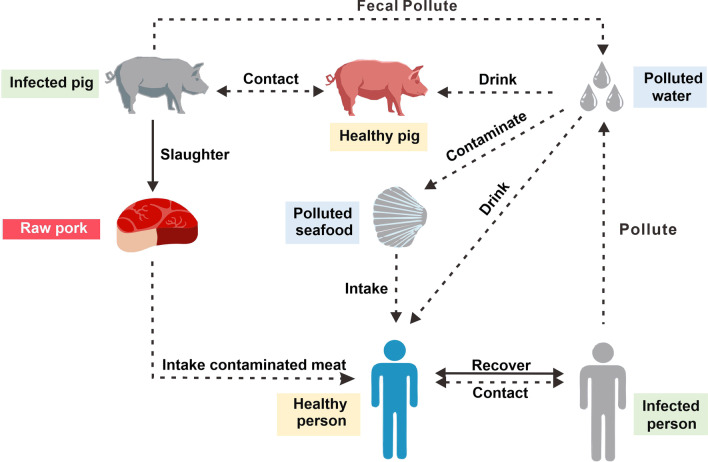
Study design of three transmission routes

This study investigated three routes of HEV transmission: person-to-person, environment-to-person, and pig-to-person. These are the known routes through which HEV-susceptible people can be infected (Fig. 1).

In the pig-to-person route, healthy pigs that drink contaminated water or come into contact with infected pigs will become infected. Infected pigs excrete faeces that then contaminate water sources, forming a cycle of pig infection. When the infected pigs are slaughtered and processed for human consumption, those working in slaughter facilities or consuming undercooked pork become infected. In the environment-to-person route, contaminated water contaminates seafood, which is then consumed by people. Contaminated water may also enter the drinking water system directly or indirectly and then be consumed by people. In the person-to-person route, infected people can infect healthy people through direct contact. Infected people can become healthy again through self-healing or after treatment.

**Fig. 2:**
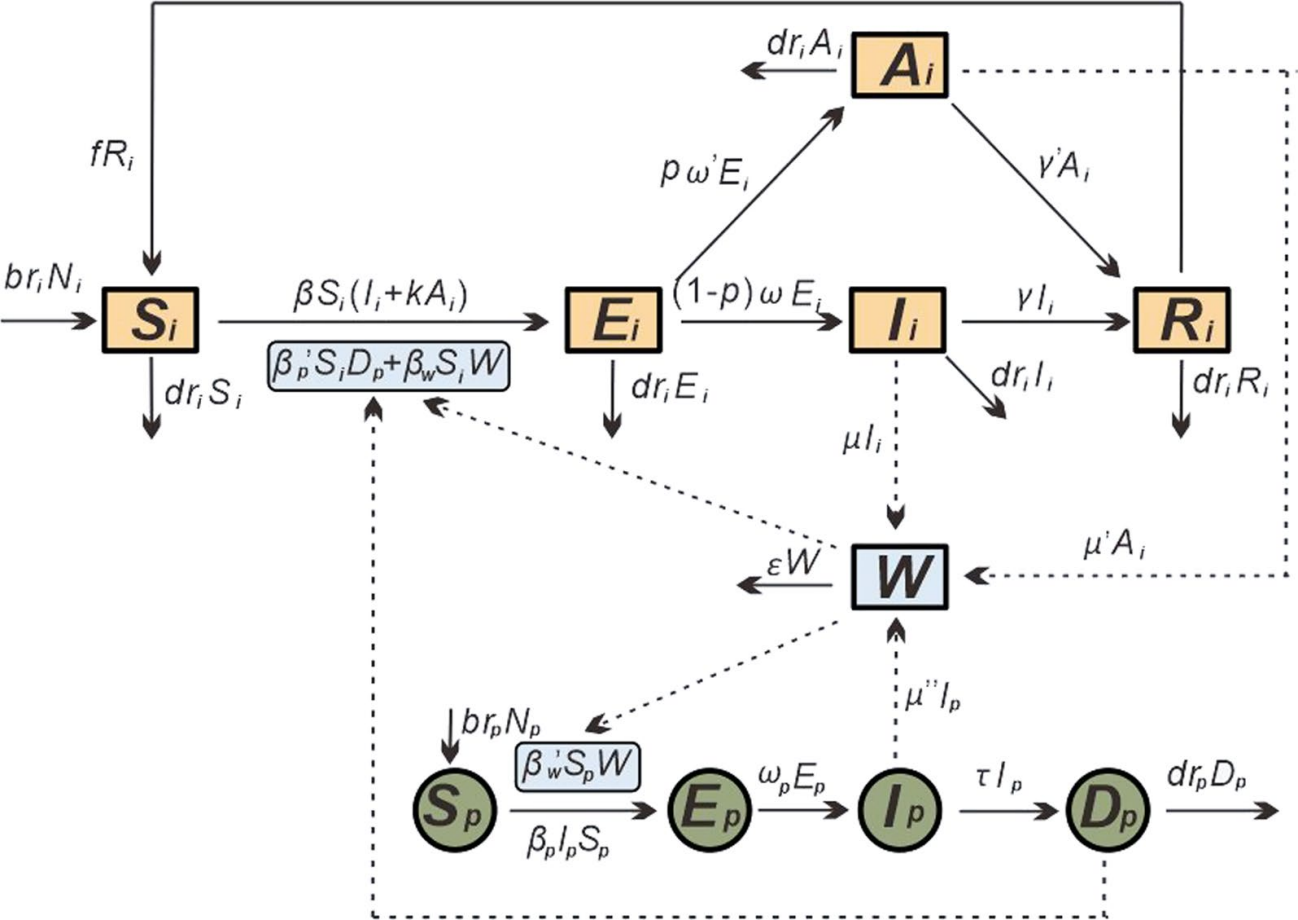
Establishing the transmission dynamics of the MHMRTDM model of hepatitis E

In the MHMRTDM, we adopted the subscript *i* and *p* to represent individuals and pigs, respectively. Person-to-person transmission includes the following: susceptible person (*S*_*i*_), exposed person (*E*_*i*_), infectious person (*I*_*i*_), asymptomatic person (*A*_*i*_), and recovered person (*R*_*i*_). Environment-to-person transmission is mainly water (*W*) transmission. The pig-to-person transmission route includes the following: susceptible pig (*S*_*p*_), exposed pig (*E*_*p*_), infectious pig (*I*_*p*_), and dead pig (*D*_*p*_). (Model 1 in Fig. 2). The definitions of each compartment are shown in Table 1.

**Table 1:** Variables definition table of the MHMRTDM

Variable	Description	Unit
S_i_	Susceptible individual density	Individuals·km^−2^
E_i_	Exposed individual density	Individuals·km^−2^
I_i_	Infectious individual density	Individuals·km^−2^
A_i_	Asymptomatic individual density	Individuals·km^−2^
R_i_	Recovered/removed individual density	Individuals·km^−2^
W	Pathogen concentration in water reservoir	Cells·ml^−3^
N_i_	Total population density	Individuals·km^−2^
N_p_	Total pig herd density	Pigs·km^−2^
S_p_	Susceptible pig density	Pigs·km^−2^
E_p_	Exposed pig density	Pigs·km^−2^
I_p_	Infectious pig density	Pigs·km^−2^
D_p_	Slaughtered pig density	Pigs·km^−2^

The model was based on the following assumptions:

- Considering the natural birth and death factors of the population and assuming the disease will not spread vertically, all new individuals born in various groups are susceptible. The population birth rate is *br*_*i*_, and the natural death rate is *dr*_*i*_.
- A susceptible person is infected through the “person-to-person”, “environment-to-person”, and “pig-to-person” routes and the infection rates are *β*, *β*_*w*_, and *β*_*p*_′, respectively. At the same time, it was assumed that the infection rate of an asymptomatic person is *k* times that of an infected person, where 0 ≤ *k* ≤ 1.
- The incubation period of an exposed person (*E*) is 1/*ω.* In the model, the changed rate from *E*_*i*_ to *A*_*i*_ was assumed to be equal to that of *I*_*i*_. Meanwhile, *p* was defined as the proportion of asymptomatic people. The rate (per day) at which an exposed person became infected was (1 − *p*)*ωE*_*i*_ (0 ≤ *p* ≤ 1), and the rate at which they acquired an asymptomatic infection was *pωE*_*i*_.
- Infected and asymptomatic people will recover after an infection period of 1/*γ* and 1/*γ*′, respectively. According to Chinese hepatitis E epidemic data released by the Ministry of Health in 2019, hepatitis E has a low case fatality rate. We therefore did not include the case fatality rate in the model for this study. The antibodies produced by a recovered person can only last for a certain period of time (antibody maintenance period, 1/*f*); thereafter, *R*_*i*_ becomes *S*_*i*_ again.
- An asymptomatic person (*A*_*i*_), infected person (*I*_*i*_), and pig (*I*_*p*_) can all excrete HEV, and it is assumed that the coefficient of HEV excretion is *μ*, *μ*′, *and μ*″, respectively. Previous studies showed that infected people present with diarrhea, whereas infected pigs remain asymptomatic. We assumed that the ability to detoxify while presenting symptoms is twice as great as the ability to detoxify while remaining asymptoms; thus, *μ* = *2μ*′ = 2*μ*″.
- The HEV will cease to exist after a certain period in the environment, and the survival time is 1/*ε*.
- In this study, we assigned the pig birth rate as *br*_*p*_ and the pig natural mortality rate as *dr*_*p*_.
- A susceptible pig is infected through "pig-to-pig" or "environment-to-pig" routes, and the infection rates are *β*_*p*_ and *β*_*w*_′, respectively.
- The incubation period of the exposed pig (*E*_*p*_) is 1/*ω*_*p*_, and the time from pig infection to slaughter is 1/$\tau$. Most people are infected with HEV after ingesting undercooked pork. A slaughtered infected pig becomes meat (pork) after a certain period of time depending on the rate of slaughtering.
- It was assumed that the number of pigs in the Jiangsu Province market is a dynamic balance, and the birth rate and death rates of pigs are the equal. Owing to infection, disease, recovery, and other reasons, several groups of people and pigs change dynamically at a certain speed, and the virus in the environment also changes dynamically over time.

The equations of the model containing the three transmission routes are as follows:

$$\frac{d{S}_{i}}{dt}=b{r}_{i}{N}_{i}+f{R}_{i}-{dr}_{i}{S}_{i}-{\beta S}_{i}\left({I}_{i}+{kA}_{i}\right)-{\beta }_{w}{S}_{i}W-{\beta }_{p}^{{\text{'}}}{S}_{i}{D}_{p}$$

$$\frac{d{E}_{i}}{dt}=\beta {S}_{i}\left({I}_{i}+k{A}_{i}\right)+{\beta }_{w}{S}_{i}W+{\beta }_{p}^{\text{'}}{S}_{i}{D}_{p}-(1-p)\omega {E}_{i}-p{\omega }^{\text{'}}{E}_{i}-{dr}_{i}{E}_{i}$$

$$\frac{d{I}_{i}}{dt}=(1-p)\omega {E}_{i}-(\gamma +{dr}_{i}){I}_{i}$$

$$\frac{d{A}_{i}}{dt}=p{\omega }^{{\text{'}}}{E}_{i}-({\gamma }^{{\text{'}}}+{dr}_{i}){A}_{i}$$

$$\frac{d{R}_{i}}{dt}=\gamma {I}_{i}+{\gamma }^{{{'}}}{A}_{i}-(f+{dr}_{i}){R}_{i}$$

$$\frac{dW}{dt}=\mu {I}_{i}+{\mu}^{{{'}}}{A}_{i}+{\mu }^{{{''}}}{I}_{p}-\varepsilon W$$

$$\frac{d{S}_{p}}{dt}=b{r}_{p}{N}_{p}-{\beta }_{w}^{{\text{'}}}{S}_{p}W-{\beta }_{p}{I}_{p}{S}_{p}$$

$$\frac{d{E}_{p}}{dt}={\beta }_{w}^{{\text{'}}}{S}_{p}W+{\beta }_{p}{I}_{p}{S}_{p}-{\omega }_{p}{E}_{p}$$

$$\frac{d{I}_{p}}{dt}={\omega }_{p}{E}_{p}-{\tau I}_{p}$$

$$\frac{d{D}_{p}}{dt}={\tau I}_{p}-{dr}_{p}{D}_{p}$$

To consistently and easily calculate the dimensions across all groups of people and modes and reduce the number of parameters, the variables and parameters of the model were normalised, so that *s*_*i*_ = *S*_*i*_/*N*_*i*_, *e*_*i*_ = *E*_*i*_/*N*_*i*_, *i*_*i*_ = *I*_*i*_/*N*_*i*_, *a*_*i*_ = *A*_*i*_/*N*_*i*_, *r*_*i*_ = *R*_*i*_/*N*_*i*_, *w* = *εW*/(*μN* + *μ″N*_*p*_), *b* = *βN*_*i*_, *b*_*W*_ = *μβ*_*W*_* N*_*i*_/*ε*, *μ* = *μ′, s*_*p*_ = *S*_*p*_/*N*_*p*_, *e*_*p*_ = *E*_*p*_/*N*_*p*_, *i*_*p*_ = *I*_*p*_/*N*_*p*_, *d*_*p*_ = *D*_*p*_/*N*_*p*_, *x* = *N*_*p*_/*N*_*i*_, *σ* = *b*_*w*_*′w* + *b*_*p*_*i*_*p*_, *b*_*p*_ = *β*_*p*_*N*_*p*_, *b*_*w*_*′* = *μ″β*_*W*_*′N*_*p*_/*ε*.

The normalised equations are as follows:

$$\frac{{ds}_{i}}{dt}={br}_{i}+f{r}_{i}-b{s}_{i}\left({i}_{i}+k{a}_{i}\right)-{b}_{w}{s}_{i}w-{b}_{p}^{{\text{'}}}{s}_{i}{d}_{p}-{dr}_{i}{s}_{i}$$

$$\frac{{de}_{i}}{dt}=b{s}_{i}\left({i}_{i}+k{a}_{i}\right)+{b}_{w}{s}_{i}w+{b}_{p}^{{\text{'}}}{s}_{i}{d}_{p}-(1-p)\omega {e}_{i}-p{\omega }^{{\text{'}}}{e}_{i}-{dr}_{i}{e}_{i}$$

$$\frac{{di}_{i}}{dt}=(1-p)\omega {e}_{i}-\gamma {i}_{i}-{dr}_{i}{i}_{i}$$

$$\frac{{da}_{i}}{dt}=p{\omega }^{{\text{'}}}{e}_{i}-{\gamma }^{{\text{'}}}{a}_{i}-{dr}_{i}{a}_{i}$$

$$\frac{{dr_{i} }}{{dt}} = \gamma ^{\prime}a_{i} + ~\gamma i_{i} - fr_{i} - dr_{i} r_{i}$$

$$\frac{dw}{dt}=\varepsilon ({i}_{i}+{a}_{i}+2{i}_{p}-w)$$

$$\frac{{ds}_{p}}{dt}=x{br}_{p}-{\sigma s}_{p}$$

$$\frac{{de}_{p}}{dt}=\sigma {s}_{p}-{\omega }_{p}{e}_{p}$$

$$\frac{{di}_{p}}{dt}={\omega }_{p}{e}_{p}-\tau {i}_{p}$$

$$\frac{{dd}_{p}}{dt}=\tau {i}_{p}-{dr}_{p}{d}_{p}$$

We built a new model (Model 2) to estimate the vaccination effects (Fig. S1 in Additional file 2) by adding the following assumptions to Model 1:

- Since the hepatitis E vaccine in China is not compulsory, we assumed that the vaccination coefficient of the vaccine is *δ*. *V* and *G* may also be infected by the people, environment, and pigs, and the infection rates are *β*, *β*_*w*_, and *β*_*p*_′, respectively.
- The time from the absence of antibody to the production of antibody after vaccination was assumed to be 1/*φ,* and the protection rate of the vaccine was assumed to be 1-*λ*.

The equations of Model 2 are as follows:

$$\frac{d{S}_{i}}{dt}=b{r}_{i}{N}_{i}+f{R}_{i}-{dr}_{i}{S}_{i}-{\beta S}_{i}\left({I}_{i}+{kA}_{i}\right)-{\beta }_{w}{S}_{i}W-{\beta }_{p}^{{\text{'}}}{S}_{i}{D}_{p}-\delta {S}_{i}$$

$$\frac{d{E}_{i}}{dt}=\beta {S}_{i}\left({I}_{i}+k{A}_{i}\right)+{\beta }_{w}{S}_{i}W+{\beta }_{p}^{{\text{'}}}{S}_{i}{D}_{p}+{\beta }_{p}V{D}_{p}+\beta V\left({I}_{i}+k{A}_{i}\right)+{\beta }_{w}VW+(1-\lambda ){(\beta }_{p}G{D}_{p}+\beta G\left({I}_{i}+k{A}_{i}\right)+{\beta }_{w}GW)-(1-p)\omega {E}_{i}-p{\omega }^{{\text{'}}}{E}_{i}-{dr}_{i}{E}_{i}$$

$$\frac{d{I}_{i}}{dt}=(1-p)\omega {E}_{i}-(\gamma +{dr}_{i}){I}_{i}$$

$$\frac{d{A}_{i}}{dt}=p{\omega }^{{\text{'}}}{E}_{i}-({\gamma }^{{\text{'}}}+{dr}_{i}){A}_{i}$$

$$\frac{d{R}_{i}}{dt}=\gamma {I}_{i}+{\gamma }^{{\text{'}}}{A}_{i}-(f+dr){R}_{i}$$

$$\frac{dW}{dt}=\mu {I}_{i}+{\mu }^{{{'}}}{A}_{i}+{\mu }^{{{''}}}{I}_{p}-\varepsilon W$$

$$\frac{dV}{dt}=\delta {S}_{i}-{\beta }_{p}V{D}_{p}-\beta V\left({I}_{i}+k{A}_{i}\right)-{\beta }_{w}VW-{dr}_{i}V-\varphi V$$

$$\frac{{dG}}{{dt}} = \varphi V - dr_{i} G - \left( {1 - \lambda } \right)~\left( {\beta _{p} GD_{p} + \beta G\left( {I_{i} + kA_{i} } \right) + \beta _{w} GW} \right)$$

$$\frac{d{S}_{p}}{dt}=b{r}_{p}{N}_{p}-{\beta }_{w}^{{{'}}}{S}_{p}W-{\beta }_{p}{I}_{p}{S}_{p}$$

$$\frac{d{E}_{p}}{dt}={\beta }_{w}^{{\text{'}}}{S}_{p}W+{\beta }_{p}{I}_{p}{S}_{p}-{\omega }_{p}{E}_{p}$$

$$\frac{d{I}_{p}}{dt}={\omega }_{p}{E}_{p}-{\tau I}_{p}$$

$$\frac{d{D}_{p}}{dt}={\tau I}_{p}-{dr}_{p}{D}_{p}$$

### Simulating the effectiveness of interventions

This study simulates three interventions: cutting off transmission routes, shortening the infectious period, and vaccination. We forecasted the incidence of infection for 2019–2023 based on the reported incidence data in Jiangsu Province during 2005–2018 and started to implement the intervention forecast for the 2018–2023 period. In addition, to investigate the difference in the effect of interventions at provincial and municipal levels, we selected three cities in Jiangsu Province with high, moderate, and low annual average incidence rates for the same operation: Zhenjiang City, Yancheng City, and Wuxi City. We compared the differences under the following scenarios:

- Scenario 1: Cutting off the transmission routes
  1. If all patients were isolated and we set the isolation time to March, June, September, and December 2018, then *b* = 0.
  2. If we blocked the route of external transmission (contaminated food and water resources) and set the intervention time points to March, June, September, and December 2018, we only set *b*_*w*_ = 0.
  3. If the pig-to-person transmission was cut off and we set the cut off time points to March, June, September, and December 2018. At the same time, we set *b*_*p*_ = 0.
- Scenario 2: Shortening the infectious period

When an infected patient receives treatment promptly after symptoms appear, the time of treatment is shortened. The recovery period 1/*γ* and 1/*γ′* will also be shortened. In this study, the recovery/infectious period was shortened from 30 days to 27, 24, 21, 18, and 15 days, respectively. We adjusted *γ* and *γ′* after December 2018 to 1.111, 1.25, 1.429, 1.667, and 2. The other parameter settings remained unchanged.

(iii) Scenario 3: Vaccinating susceptible people

The vaccine intervention model has two more warehouses than the original model; we used *V* and *G* to denote those who were vaccinated but not resistant to infectious diseases and those who are resistant after vaccination, respectively. We then set the monthly vaccination ratio of the vaccine using a monthly vaccination coefficient, *δ*. We set *δ* to 20%, 40%, 60%, 80%, and 100%, respectively. All other parameters remained unchanged.

### Parameter estimation

The parameters of the population in the MHMRTDM used in this study were *br*_*i*_, *dr*_*i*_, *b*, *b*_*W*_, *p*, *ω*_*i*_, *γ*, *γ′*, *ε,* and *f* (Table 2). According to the 2006 Statistical Yearbook of each city in Jiangsu Province, this study used month as the basic time unit of the birth rate and natural death rate; thus, *br*_*i*_ and *dr*_*i*_ were taken as 1/12 of each city’s annual birth rate and death rates.

**Table 2:** Parameter definitions and values

Parameter	Description	Unit	Value	Range	Method
*β*	Susceptible person-to -infected person contact rate	km^2^·individuals^−1^·month^−1^	–	≥ 0	–
*b*	Scaled susceptible person-to-infected person contact rate	Month^−1^	–	≥ 0	Curve fitting
*β*_*w*_	Reservoir-to-Person contact rate	ml·cells^−1^·month^−1^	–	≥ 0	–
*b*_*w*_	Scaled Reservoir-to-Person contact rate	Month^−1^	–	≥ 0	Curve fitting
*β*_*p*_*ʹ*	Pig-to-Person contact rate	km^2^·pigs^−1^·month^−1^	–	≥ 0	–
*b*_*p*_*ʹ*	Scaled pig-to-person contact rate	Month^−1^	–	≥ 0	Curve fitting
*β*_*w*_*ʹ*	Reservoir-to-Pig contact rate	ml·cells^−1^·month^−1^	–	≥ 0	–
*b*_*w*_*ʹ*	Scaled Reservoir-to-Pig contact rate	Month^−1^	–	≥ 0	Curve fitting
*β*_*p*_	Pig-to-pig contact rate	km^2^·pigs^−1^·month^−1^	–	≥ 0	–
*b*_*p*_	Scaled Pig-to-Pig contact rate	Month^−1^	–	≥ 0	Curve fitting
*σ*	Scaled force of infection from environment-to-pig and pig-to-pig	Month^−1^	0.9	≥ 0	Reference [26]
*ω*	Incubation relative rate of individuals	Month^−1^	0.7500	0.5–2.5	Reference [25, 27]
*γ*	Recovery rate of the infectious	Month^−1^	1.0000	0.7143–1.0714	Reference [25]
*γ*′	Recovery rate of the asymptomatic	Month^−1^	1.0000	0.7143–1.0714	Reference [25]
*ε*	Pathogen lifetime relative rate	Month^−1^	1.3333	≥ 1.3333	Reference [28]
*f*	Hepatitis E antibody elimination rate	Month^−1^	0.00595	≥ 0.00595	Reference [29]
*ω*_*p*_	Incubation relative rate of pig	Month^−1^	1.0000	0–1	Reference [23]
*τ*	Slaughtered rate of infected pigs	Month^−1^	0.333	0–1	Reference [23]
*μ*	The rate of the infected person shedding the virus to the reservoir	Cells·km^2^/(cells·month·ml)	–	0–1	Assumption
*μ′*	The rate of asymptomatic person shedding the virus to the reservoir	Cells·km^2^/(cells·month·ml)	–	0–0.5	Assumption
*μʹʹ*	The rate of infected pigs shedding the virus to the reservoir	Cells·km^2^/(cells·month·ml)	–	0–0.5	Assumption
*p*	Proportion of the symptomatic	1	0.9	0–0.15	Reference [25]
*br*_*i*_	Birth rate of population	1	0.00931	0–1	Statistical Yearbook
*dr*_*i*_	Death rate of population	1	0.00702	0–1	Statistical Yearbook
*br*_*p*_	Birth rate of pig herd	1	0.00667	0–1	Reference [23]
*dr*_*p*_	Death rate of pig herd	1	0.00667	0–1	Reference [23]
*x*	Ratio of pig population density to population density	1	0.333	≥ 0	China Rural Statistical Yearbook, the main data bulletin of the sixth national census in 2010
*k*	Relative transmissibility rate of asymptomatic to symptomatic individuals	1	1	0–1	Reference [25]
*δ*	Monthly vaccination ratio	1	1	0–1	Artificial setting
*λ*	Effective rate of vaccination	1	1–0.933	0–1	Reference [29]
*φ*	Vaccination onset rate	Month^−1^	1/6	0–1	Reference [30]

–: not applicable

According to previous studies [25], during an hepatitis E epidemic, the proportion of asymptomatic infections is *p* = 0.9, the reciprocal of the average disease course is *γ* = 1, and the reciprocal of the incubation period is *ω*_*i*_ = 0.75.

An individual’s HEV IgG “antibody maintenance time” after infection with HEV exceeds 14 years [29]; thus, *f* = 1/(14 × 12) = 0.00595. Based on the literature, the survival time of HEV in water and the environment is 1/*ε*, about 3 weeks; thus, thus we set *ε* = 1.3333 [28].

The parameters of the pig groups are *br*_*p*_, *dr*_*p*_, *b*_*p*_, *ω*_*p*_, and *τ*. According to the literature [23], the time from birth to slaughter of pigs is approximately 5 months; this was used to estimate the monthly birth rate and natural mortality of pigs, namely *br*_*p*_ = 0.2 and *dr*_*p*_ = 0.2. The time from birth to infection of pigs is approximately 2 months, and the monthly slaughter rate of infected pigs is *τ* = 0.333. According to the literature, the expected value of pig HEV infectivity is between 2.68 × 10^–2^ and 3.45 × 10^–2^ per day [26]. In the previously mentioned study, the rate of infection of pigs was 3 × 10^–2^ per day, which corresponds to 0.9 per month (*b*_*p*_ = 0.9/month). According to the "China Rural Statistical Yearbook 2019" and the main data of the sixth national census in 2010, the ratio of the total number of pigs to the total population density is *x* = 0.333.

The curve fitting time of this study began in 2005. For models with intervention measures, the values of the parameters at the beginning of the intervention are the simulated output values of the model at the corresponding time without intervention. The simulated time for vaccination was 2019–2023, and we simulated with inoculation coefficients of five scenarios, which were set to 20%, 40%, 60%, 80%, and 100%.

### Seasonality estimation

Hepatitis E has obvious seasonality and periodicity; thus, it was necessary to correct the model for these factors. Based on a previous study [31], the seasonal and periodic correction results of the model can be obtained using cosine function correction $\beta$.

$$\beta \left( t \right) = \beta _{0} \left[ {{{1~ + ~\rho \cos \left( {~2\pi t} \right)} \mathord{\left/ {\vphantom {{1~ + ~\rho \cos \left( {~2\pi t} \right)} T}} \right. \kern-\nulldelimiterspace} T}} \right]$$

where *β*(*t*) represents *β* at *t* time and *β*_0_ represents *β* at the initial time of simulation, with a period of 1 year. In this study, month is the basic time unit of hepatitis E incidence; thus, it is necessary to introduce the parameter seasonal cycle *T* = 12. It was also observed that the incidence of hepatitis E in the non-epidemic season in Jiangsu Province was close to zero, indicating that it was the lowest epidemic month of the disease, that is, when *T* = 6* k* (*k* is odd), *β* (*t*) = *β*(0) (1-*ρ*) = 0, and *c* = 1. Because the sine function can be regarded as the translation of the cosine function, the sine expression was used in this study. Considering that the monthly incidence period of hepatitis E in Jiangsu Province is different from that in Changsha, we introduced the new parameter Δ*t*, under which the correction effect of Δ*t* can be divided in two parts, that is, the conversion from cosine function to sine function and the seasonal difference between Changsha and Jiangsu Provinces. The equation used in this study was as follows:

$$b~ = b_{0} \left[ {1~ + ~\sin \left( {\frac{{2\pi \left( {t + \Delta t} \right)}}{{12}}} \right)} \right]$$

$$b_{w} ~ = b_{{w0}} \left[ {1~ + ~\sin \left( {\frac{{2\pi \left( {t + \Delta t} \right)}}{{12}}} \right)} \right]$$

Among them, Δ*t* is set and adjusted in sections according to the principle of best fit.

### Evaluating the effectiveness of interventions

Measures to assess the effectiveness of interventions were used in this study with a total attack rate (*TAR*). The formula is as follows:

$$TAR = \frac{{TN}}{N} \times 100\%$$

In the equation, *TN* and *N* refer to the total number of new cases and the total population number, respectively.

### Simulation method and statistical analysis

In this study, Microsoft Excel (2020 version; Microsoft Corp., Redmond, WA) was used for the entry and management of related data and related mapping, and Berkeley Madonna 8.3.18 software (developed by Robert Macey and George Oster of the University of California at Berkeley, CA, USA) was used for modelling, while the Runge–Kutta method of order 4 with tolerance set at 0.001 was used to perform curve fitting. When curve fit is in progress, Berkeley Madonna displays the root mean square deviation between the data and best run so far [32, 33]. The IBM SPSS Statistics for Windows, (version 23.0; JBM Corp., Armonk, NY) curve estimation function was used to obtain *R*^2^ to describe the goodness of fit.

### Sensitivity analysis

Sensitivity analysis was conducted for 14 parameters, which were divided into 42 values according to their range. In this study, sensitivity analysis was performed for the 2004–2018 date of Jiangsu Province. In general, the *TAR* of infectious diseases has different rise and decline trends. Each parameter was divided into three segments and calibrated with a minimum, moderate, and maximum or greater segment when it did not have a maximum.

## Results

### Epidemiological characteristics

**Fig. 3:**
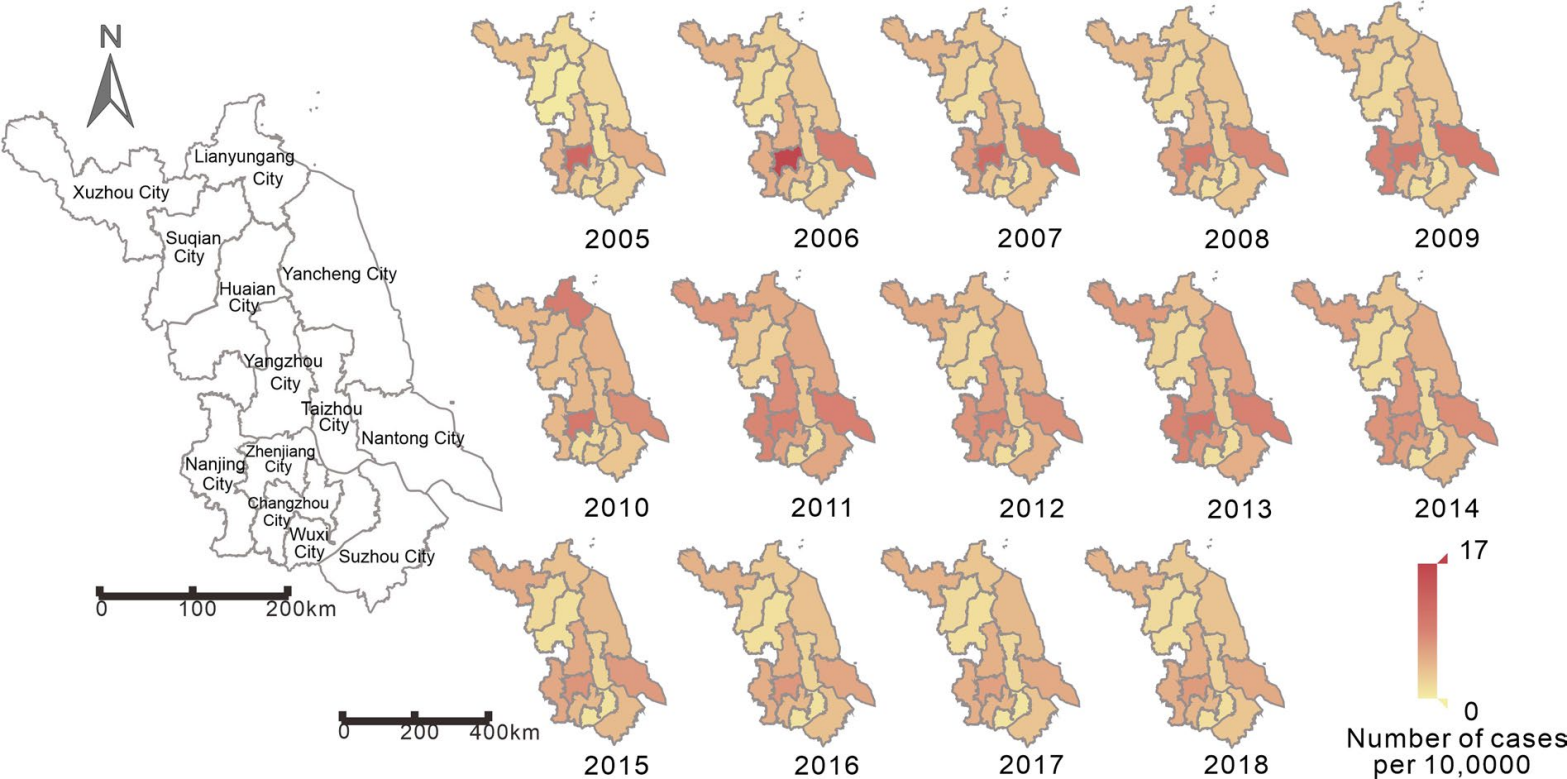
Incidence map of each city in Jiangsu Province from 2005 to 2018

**Fig. 4:**
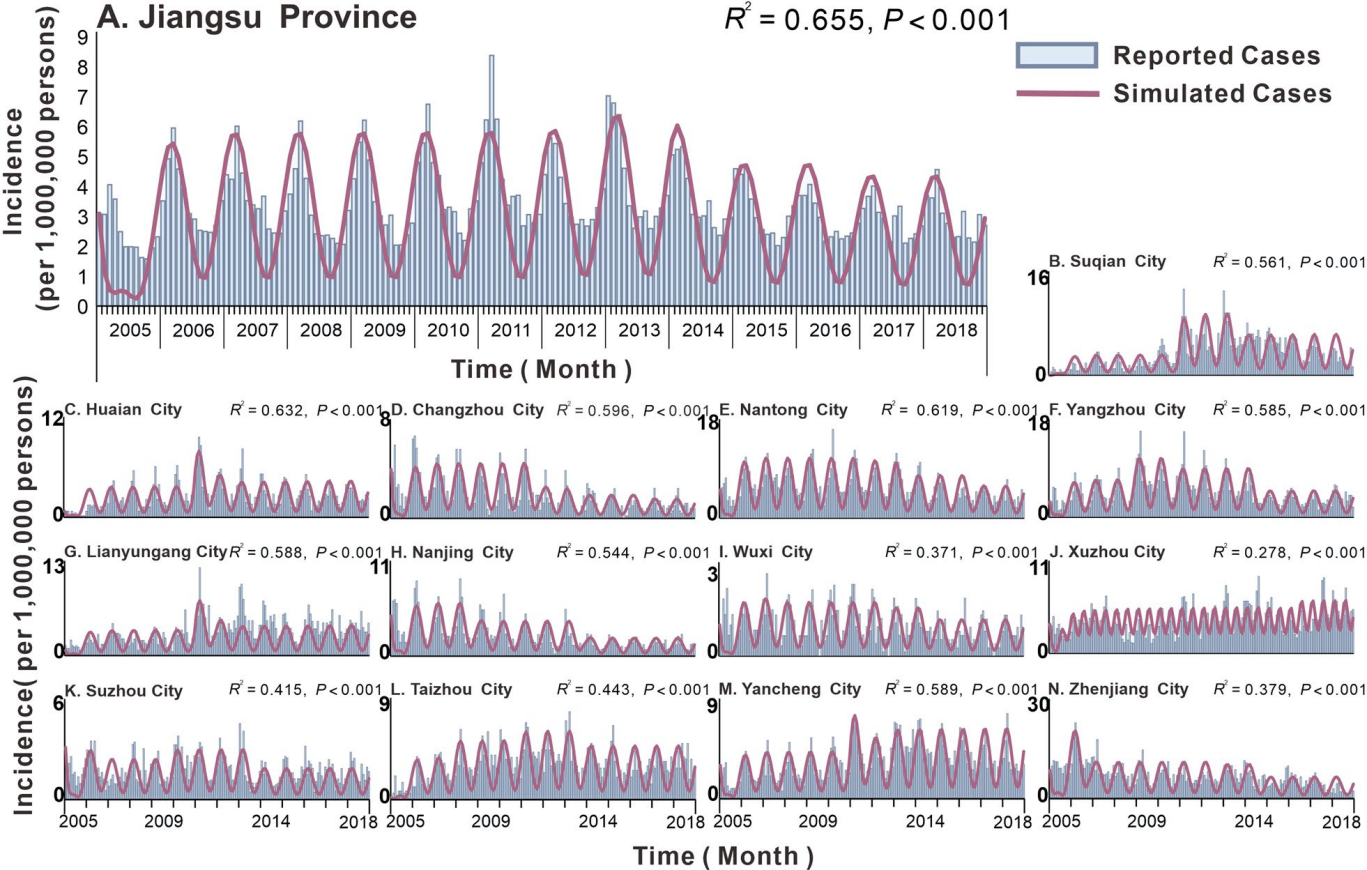
Simulated situation of hepatitis E incidence in Jiangsu Province and other cities during 2005–2018. **A** Jiangsu Province; **B** Suqian City; **C** Huaian City; **D** Changzhou City; **E** Nantong City; **F** Yangzhou City; **G** Lianyungang City; **H** Nanjing City; **I** Wuxi City; **J** Xuzhou City; **K** Suzhou City; **L** Taizhou City; **M** Yancheng City; **N** Zhenjiang City

A total of 44 923 cases of hepatitis E were reported in Jiangsu Province, China, from 2005 to 2018 (average annual incidence of 4.12 per 100 000 people). The annual incidence of hepatitis E in Jiangsu Province peaked in 2007 (4.35 per 100 000 people), 2011 (5.21 per 100 000 people), and 2013 (4.94 per 100 000 people). The lowest incidence was 2.96 per 100 000 people in 2005. The three cities with the highest average annual incidence in Jiangsu Province (Fig. 3) were Zhenjiang City (7.90 per 100 000 people), Nantong City (6.96 per 100 000 people), and Yangzhou City (5.72 per 100 000 people). The three cities with the lowest average annual incidence were Wuxi City (1.32 per 100 000 people), Suzhou City (2.24 per 100 000 people), and Changzhou City (2.32 per 100 000 people). The annual incidence rates in Lianyungang City (4.37 per 100 000 people) and Taizhou City (4.06 per 100 000 people) are increasing each year, while the annual incidence rates in Changzhou City (2.32 per 100 000 people) and Nanjing City (3.07 per 100 000 people) are decreasing each year. The overall annual incidence rates in other regions showed an upward trend first, followed by a downward trend. The peak incidence rate of hepatitis E in Jiangsu Province and other cities was around March; however, the peak transmissibility was in December and January (Table 3). The results of curve fitting showed that the MHMRTDM fits the data well (Fig. 4). Jiangsu Province (*R*^2^ = 0.655, *P* < 0.001) and the first three cities that fit well are Huai’an City (*R*^2^ = 0.632, *P* < 0.001), Nantong City (*R*^2^ = 0.619, *P* < 0.001), and Changzhou City (*R*^2^ = 0.596, *P* < 0.001).

**Table 3:** Peak time and transmission capacity of different transmission routes of hepatitis E in Jiangsu Province

Area	*b*_*i*_	*b*_*w*_	*b*_*p*_′	Transmissibility peak time (month)	Incidence peak time (month)
Jiangsu Province	4.32 × 10^–10^	1.02 × 10^–7^	9.31 × 10^–5^	12 and 1	3
Changzhou City	1.23 × 10^–9^	1.17 × 10^–7^	4.76 × 10^–5^	12	2
Huaian City	8.23 × 10^–10^	8.03 × 10^–8^	8.12 × 10^–5^	12 and 1	3
Suzhou City	4.76 × 10^–18^	4.35 × 10^–10^	4.28 × 10^–5^	12 and 1	2
Nantong City	9.96 × 10^–10^	2.22 × 10^–7^	1.59 × 10^–4^	12	3
Lianyungang City	8.23 × 10^–10^	8.03 × 10^–8^	8.12 × 10^–5^	12 and 1	3
Yancheng City	7.48 × 10^–10^	1.11 × 10^–7^	9.68 × 10^–5^	12 and 1	3
Yangzhou City	4.76 × 10^–18^	4.35 × 10^–10^	4.28 × 10^–5^	12	3
Zhenjiang City	1.99 × 10^–10^	5.63 × 10^–8^	1.49 × 10^–4^	12 and 1	3
Taizhou City	9.35 × 10^–10^	1.02 × 10^–7^	9.73 × 10^–5^	1	3
Suqian City	6.37 × 10^–10^	8.04 × 10^–8^	8.19 × 10^–5^	1	3
Wuxi City	1.19 × 10^–9^	9.79 × 10^–8^	3.11 × 10^–5^	12	2.75
Xuzhou City	6.29 × 10^–11^	1.02 × 10^–8^	1.21 × 10^–4^	6 and 12	3 and 8
Nanjing City	8.47 × 10^–10^	1.01 × 10^–7^	6.51 × 10^–5^	1	3

### Effectiveness of cutting off transmission routes

**Fig. 5:**
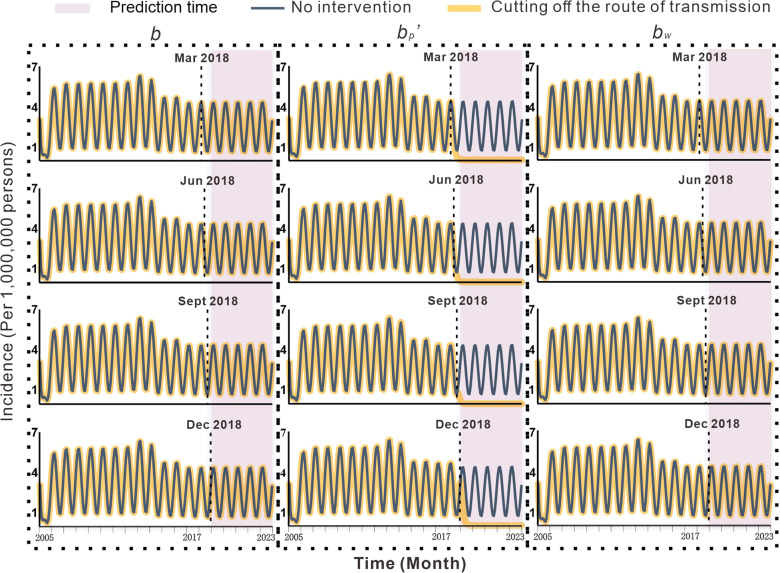
Effects of cutting the transmission routes on the incidence of hepatitis E

As shown in Fig. 5, the results of cutting off the person-to-person and environment-to-person routes in Jiangsu Province are not obvious regarding prevention and control. Cutting off the pig-to-person route, however, can effectively control the spread of the diseases. If the pig-to-person transmission route was cut off in March 2018, the *TAR* from March 2018 to December 2023 was predicted to be 1.398 per 100 000 people, which was lower than the predicted *TAR* of 17.437 per 100 000 people (reduction of 92.0%). If the transmission route was cut off in June 2018, the *TAR* from June 2018 to December 2023 was 0.504 per 100 000 people, which is lower than the predicted *TAR* of 16.310 per 100 000 people (reduction of 96.9%). If the transmission route was cut off in September 2018, the *TAR* from September 2018 to December 2023 was 0.301 per 100 000 people, which was lower than the predicted *TAR* of 15.895 per 100 000 people (reduction of 98.1%). If the transmission route was cut off in December 2018, the *TAR* between December 2018 and December 2023 was 1.109 per 100 000 people, which is lower than the predicted *TAR* of 15.508 per 100 000 people (reduction of 92.9%). The effectiveness of cutting off transmission routes in Zhenjiang City, Yancheng City, and Wuxi City was shown are shown in Additional file 1: Table S1 and Additional file 2: Figs. S2–S4.

### Effectiveness of shortening the infectious period

**Fig. 6:**
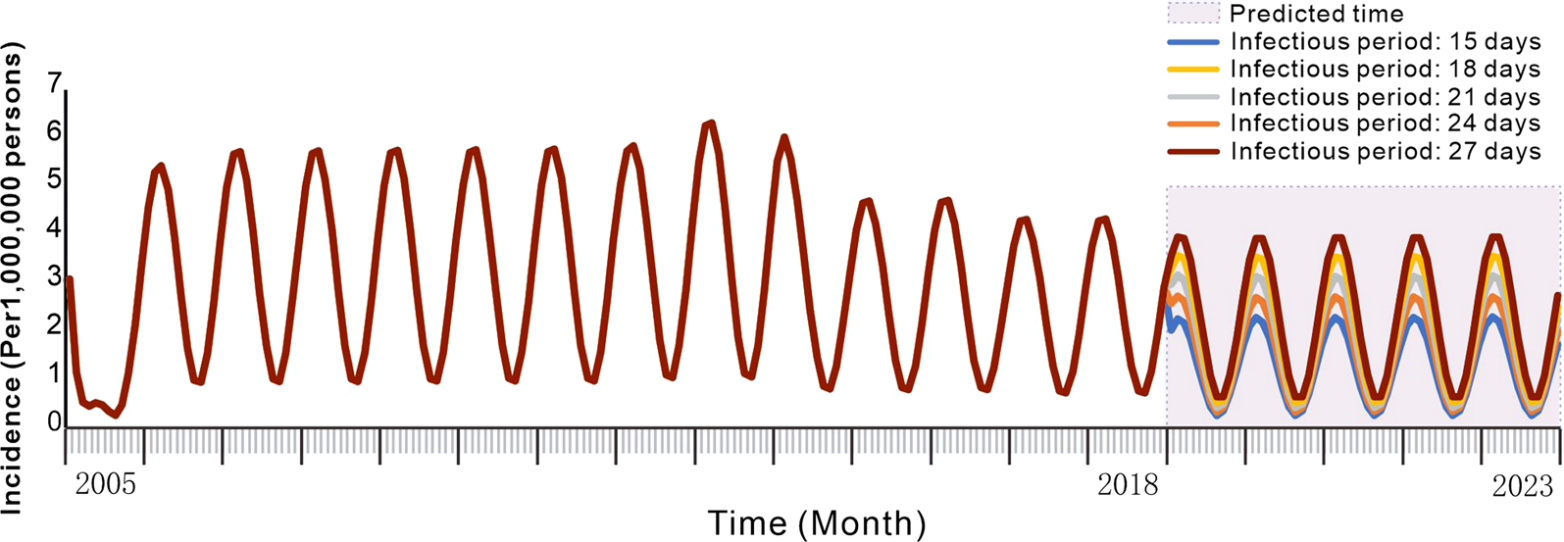
Simulation of shortening the incidence of hepatitis E infection

The incidence of hepatitis E in Jiangsu Province was reduced when the infectious period was shortened (Fig. 6). When the predicted infection period was 27, 24, 21, 18, and 15 days without intervention from 2019 to 2023, the *TAR* values (and reduction proportions) per 100 000 people were 13.721 (9.8%), 12.195 (19.9%), 10.666 (29.9%), 9.130 (40.0%), and 7.601 (50.1%), respectively. The effectiveness of shortening the infectious period in Zhenjiang City, Yancheng City, and Wuxi City are shown in the Additional file 1: Table S1 and Additional file 2: Figs. S5–S7.

### Effectiveness of assessing the effectiveness of vaccination

**Fig. 7:**
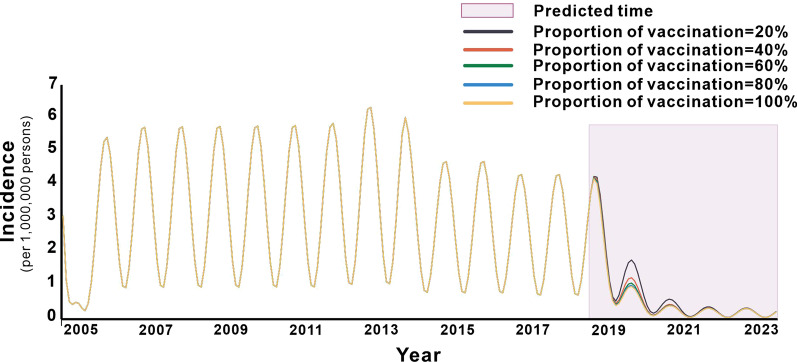
Effects of different vaccination coefficients on the incidence of hepatitis E infection

As shown in Fig. 7, the simulation of general population immunisation in Jiangsu Province decreased the *TAR* after vaccination and the vaccine effect increased with the vaccination coefficient. Within five years of the simulated 2019–2023 period, when the vaccination coefficient was 0.2, the *TAR* was 4.496/100 000, showing a decrease of 70.5% compared with that without intervention. Similarly, vaccination coefficients of 0.4, 0.6, 0.8, and 1 yielded *TAR* values per 100 000 people (and decreasing proportions) of 3.932 (74.2%), 3.754 (75.3%), 3.667 (75.9%), and 3.614 (76.3%), respectively. The effectiveness of vaccination in Zhenjiang City, Yancheng City, and Wuxi City are shown in the Additional file 1: Table S1 and Additional file 2: Figs. S8–S10.

### Sensitivity analysis

In this study, all parameters executed in the model are consistent with the range of reference values. The MHMRTDM was sensitive to *p*, *γ*, and *x*, but not sensitive to other parameters. (Additional file 2: Fig. S11).

## Discussion

The World Health Organization has formulated a plan called “Hepatitis Elimination in 2030”, but owing to its complex transmission, only few mathematical model studies have focused on HEV transmission, such as the SIR model and the Susceptible-Exposed-Symptomatic-Asymptomatic-Recovered/Removed-Water (SEIARW) model [24, 25]. Therefore, this study first clarified the transmission mechanism of how the virus transmits through multiple hosts and the environment ("person-to-person", "environment-to-person", and "pig-to-person") and then explored the influences of meteorological factors on the disease incidence. These factors are important for the prevention and control of hepatitis E.

According to the determination coefficient (*R*^2^) of the goodness of fit test, the model fits the reported incidence in different cities in Jiangsu Province well, indicating that the model is suitable for this study. At the same time, the effectiveness of the model fitting is consistent with the multi-pathway HEV SEIARW transmission model and its application in Changsha city [25].

In recent years, the annual incidence of hepatitis E in China has been rising. From 2004 to 2017, the annual incidence of hepatitis E in China increased from 1.27/100 000 to 2.10/100 000[34]. The average annual incidence of hepatitis E in Jiangsu Province from 2005 to 2018 was 4.12/100 000, which was significantly higher than the national level. However, from 2013 to 2018, the incidence of hepatitis E in Jiangsu Province displayed a downward trend, possibly owing to the strengthened prevention and control measures of the local CDC or due to vaccination. In 2011, the world’s first hepatitis E vaccine (HEV 239, Xiamen Innovax Biotech, Xiamen, China) was licensed in China. Volunteers were recruited in Jiangsu Province for the vaccine study, which was a large clinical test involving 120 000 participants [35].

At the same time, our results indicated that the incidence of hepatitis E varies from region to region. According to the Statistical Yearbook of Jiangsu Province, the gross domestic product rankings of Zhenjiang City and Yangzhou City are relatively low, while those of Suzhou City, Wuxi City, and Changzhou City rankings are high Economic development is likely to be an important reason for the uneven distribution of disease burden in Jiangsu Province. Additionally, climatic environment, population, urban environment, and human factors may represent additional reasons for the uneven disease burden distribution in Jiangsu Province.

The peak time of the disease in Jiangsu Province and its cities is in February and March each year, which is consistent with a previous study [36]. This period coincides with the Chinese Lunar New Year [22]. Perhaps because there are more holidays during the Chinese New Year than usual and people eat out and socialise more, the number of people going out for social activities increases. The greater demand for food will therefore also increase the purchase of poultry, livestock (such as pigs), and seafood; thus, these two factors probably increase transmission rates. Concurrently, the months of February and March are relatively cold, which may lower people’s immunity and consequently increase their susceptibility to the virus. Therefore, health education regarding food quality control and personal hygiene should be strengthened during this period. However, we found that transmissibility usually peaked in December and January, 3 months before the peak incidence of hepatitis E; this also coincides with the time pigs grow up and become infected with the virus, which can then be transmitted to people.

The peak of the disease in each city of Jiangsu Province is mainly in March, while the peak in August is very small (not included when fitting the model). However, the peak of rainfall is in August, which is consistent with the second incidence peak of the incidence, indicating that the incidence of the disease in Jiangsu Province is mainly related to the pig-to-person transmission route, but not closely related to the environment-to-person transmission route.

According to the model’s prediction, without intervention, the incidence will show continual cyclical changes and the epidemic will continue to develop without a spontaneous end. Intervention effects at provincial and municipal levels in Jiangsu Province were very similar. When the person-to-person and environment-to-person transmission routes were cut off, the incidence hardly decreased; conversely, cutting off the pig-to-person transmission route has achieved significant results. This indicates that controlling the contact between pigs and contaminated water as well as the quality of pork and corresponding meat products in the market can effectively control the transmission of HEV. Residents should ensure that they only consume pork meat that has been properly and thoroughly cooked, and this precaution can result in improved eating habits of the residents. The gross domestic product ranking of Jiangsu Province is among the highest in China (Statistical Yearbook of China 2019), and many related studies have indicated that HEV is mainly contracted from food in areas with better economic development [7–9].

Furthermore, the results showed that shortening the period of infection can lower the incidence of HEV, representing another effective intervention and suggesting that we should aim for early detection, early diagnosis, and early treatment for controlling HEV transmission. To achieve this, it is necessary to improve the detection rate and treatment of hepatitis E. In addition, a study has shown that the hepatitis E vaccine has a good effect on controlling the disease [29], and our study also showed that the higher the vaccination coefficient, the more the incidence rate decreases. When the vaccination coefficient was 60%, the incidence has already decreased even more significantly. When the vaccination rate reached 80%, the disease was effectively under control. However, this level of vaccination coefficient is very difficult to achieve because (1) only those 16 years and older are eligible for vaccination. However, according to the Statistical Yearbook of Jiangsu Province in 2018, 14.7% of the population was aged 0–14 years; and (2) the vaccination is voluntary and is self-limited for most people; thus, the vaccination rate will likely remain low. However, given that hepatitis E has a mortality rate of 1–3% in people aged 14–40 years [37] and up to 30% in pregnant women [38], and an even worse effect on those with underlying hepatitis [39], vaccination is necessary.

This study has some limitations. We used secondary information, mainly from the Statistical Yearbook of Jiangsu Province, and the key parameters *p*, *γ*, and *x* used in our model were derived from references instead of first-hand data. This may have influenced the accuracy of the model. The model should be further adjusted according to specific parameters for each city. In addition, further studies that model a larger variety of hepatitis E interventions and explore different variable factors, such as meteorological data, would be useful.

## Conclusions

In Jiangsu Province, the main route of HEV transmission is from pigs to humans in Jiangsu Province. Effective prevention measures for hepatitis E are needed to control the transmission from pigs to humans, strengthen the management of contact between pigs and contaminated water, and improve the quality control of market pork and related meat products. In addition, effective treatment and hepatitis E vaccination can also effectively prevent the spread of this disease.

## Supplementary Information

**Additional file 1: Table S1.** Evaluates the effectiveness of intervention in 3 cities of Jiangsu Province, China.

**Additional file 2: Figure S1.** Establishing the transmission dynamics of the vaccination intervention model of hepatitis E. **Figure S2.** Effects of cutting the transmission routes on the incidence of hepatitis E of Zhenjiang City. **Figure S3.** Effects of cutting the transmission routes on the incidence of hepatitis E of Yancheng City. **Figure S4.** Effects of cutting the transmission routes on the incidence of hepatitis E of Wuxi City. **Figure S5.** Simulation of shortening the incidence of hepatitis E infection of Zhenjiang City. **Figure S6.** Simulation of shortening the incidence of hepatitis E infection of Yancheng City. **Figure S7.** Simulation of shortening the incidence of hepatitis E infection of Wuxi City. **Figure S8.** Effects of different vaccination coefficients on the incidence of hepatitis E infection of Zhenjiang City. **Figure S9.** Effects of different vaccination coefficients on the incidence of hepatitis E infection of Yancheng City. **Figure S10.** Effects of different vaccination coefficients on the incidence of hepatitis E infection of Wuxi City. **Figure S11.** The sensitivity analysis of parameter.

## Data Availability

Data supporting the conclusions of this article are included within the article.

## Abbreviations

HEV: Hepatitis E virus
HEV-1: Genotype 1 of hepatitis E virus
HEV-2: Genotype 2 of hepatitis E virus
HEV-3: Genotype 3 of hepatitis E virus
HEV-4: Genotype 4 of hepatitis E virus
HIV: Human immunodeficiency virus
AIDS: Acquired immune deficiency syndrome
ARIMA: Autoregressive integrated moving average (model)
SIR: Susceptible–infectious–recovered (model)
MHMRTDM: Multi-host and multi-route transmission dynamic model
IgG: Immunoglobin G
*TAR*: Total attack rate
*R*^2^: Coefficient of determination
SEIARW: Susceptible–exposed–symptomatic–asymptomatic–recovered/removed–water (model)
