# Supplementary material for: Feasibility of controlling hepatitis E in Jiangsu Province, China: a modelling study
Source: Infect Dis Poverty. 2021 Jun 29;10:91. doi: 10.1186/s40249-021-00873-w (PMC8240442; doi:10.1186/s40249-021-00873-w)
Supplement: Supplementary file 1 — Additional file 1: Table S1. Evaluates the effectiveness of intervention in 3 cities of Jiangsu Province, China. [file 40249_2021_873_MOESM1_ESM.docx]

**Table S1** **Evaluates the effectiveness of intervention in 3 citys of Jiangsu Province**

1. **Zhenjiang City**

| Zhenjiang City | Average annual incidence of 7.90 per 100,000 people | | | |
| --- | --- | --- | --- | --- |
|  |  |  | TAR（per 100,000 people） | Reduction |
|  | Cutting off the person-to-person route | Mar/18 | 1.982 | 92.83% |
|  |  | Jun/18 | 0.768 | 97.03% |
|  |  | Sep/18 | 0.425 | 98.31% |
|  |  | Dec/18 | 1.642 | 93.32% |
|  | Normal infectious period | 30days | 24.120 | - |
|  | Shorten the duration of infection | 27days | 21.733 | 9.90% |
|  |  | 24days | 19.332 | 19.85% |
|  |  | 21days | 16.919 | 29.85% |
|  |  | 18days | 14.499 | 39.89% |
|  |  | 15days | 12.046 | 50.06% |
|  | Vaccination | Vaccination coefficient = 20% | 7.116 | 70.50% |
|  |  | Vaccination coefficient = 40% | 6.223 | 74.20% |
|  |  | Vaccination coefficient = 60% | 5.943 | 75.36% |
|  |  | Vaccination coefficient = 80% | 5.805 | 75.93% |
|  |  | Vaccination coefficient = 100% | 5.720 | 76.28% |

1. **Yancheng City**

| Yancheng City | Average annual incidence of 4.33 per 100,000 people | | | |
| --- | --- | --- | --- | --- |
|  |  |  | TAR（per 100,000 people） | Reduction |
|  | Cutting off the person-to-person route | Mar/18 | 1.933 | 92.84% |
|  |  | Jun/18 | 0.752 | 97.02% |
|  |  | Sep/18 | 0.418 | 98.30% |
|  |  | Dec/18 | 1.602 | 93.33% |
|  | Normal infectious period | 30days | 23.554 | - |
|  | Shorten the duration of infection | 27days | 21.221 | 9.90% |
|  |  | 24days | 18.877 | 19.85% |
|  |  | 21days | 16.521 | 29.86% |
|  |  | 18days | 14.158 | 39.89% |
|  |  | 15days | 11.763 | 50.06% |
|  | Vaccination | Vaccination coefficient = 20% | 6.946 | 70.51% |
|  |  | Vaccination coefficient = 40% | 6.073 | 74.22% |
|  |  | Vaccination coefficient = 60% | 5.800 | 75.38% |
|  |  | Vaccination coefficient = 80% | 5.665 | 75.95% |
|  |  | Vaccination coefficient = 100% | 5.582 | 76.30% |

1. **Wuxi City**

| Wuxi City | Average annual incidence of 1.32 per 100,000 people | | | |
| --- | --- | --- | --- | --- |
|  |  |  | TAR（per 100,000 people） | Reduction |
|  | Cutting off the person-to-person route | Mar/18 | 0.379 | 93.00% |
|  |  | Jun/18 | 0.133 | 97.39% |
|  |  | Sep/18 | 0.103 | 97.93% |
|  |  | Dec/18 | 0.350 | 92.76% |
|  | Normal infectious period | 30days | 4.733 | - |
|  | Shorten the duration of infection | 27days | 4.265 | 9.89% |
|  |  | 24days | 3.794 | 19.84% |
|  |  | 21days | 3.321 | 29.84% |
|  |  | 18days | 2.846 | 39.88% |
|  |  | 15days | 2.364 | 50.06% |
|  | Vaccination | Vaccination coefficient = 20% | 1.386 | 70.72% |
|  |  | Vaccination coefficient = 40% | 1.210 | 74.43% |
|  |  | Vaccination coefficient = 60% | 1.156 | 75.58% |
|  |  | Vaccination coefficient = 80% | 1.130 | 76.14% |
|  |  | Vaccination coefficient = 100% | 1.113 | 76.48% |
