# Supplementary material for: Feasibility of controlling hepatitis E in Jiangsu Province, China: a modelling study
Source: Infect Dis Poverty. 2021 Jun 29;10:91. doi: 10.1186/s40249-021-00873-w (PMC8240442; doi:10.1186/s40249-021-00873-w)
Supplement: Supplementary file 2 — Additional file 2: Figure S1. Establishing the transmission dynamics of the vaccination intervention model of hepatitis E. Figure S2. Effects of cutting the transmission routes on the incidence of hepatitis E of Zhenjiang City. Figure S3. Effects of cutting the transmission routes on the incidence of hepatitis E of Yancheng City. Figure S4. Effects of cutting the transmission routes on the incidence of hepatitis E of Wuxi City. Figure S5. Simulation of shortening the incidence of hepatitis E infection of Zhenjiang City. Figure S6. Simulation of shortening the incidence of hepatitis E infection of Yancheng City. Figure S7. Simulation of shortening the incidence of hepatitis E infection of Wuxi City. Figure S8. Effects of different vaccination coefficients on the incidence of hepatitis E infection of Zhenjiang City. Figure S9. Effects of different vaccination coefficients on the incidence of hepatitis E infection of Yancheng City. Figure S10. Effects of different vaccination coefficients on the incidence of hepatitis E infection of Wuxi City. Figure S11. The sensitivity analysis of parameter. [file 40249_2021_873_MOESM2_ESM.docx]

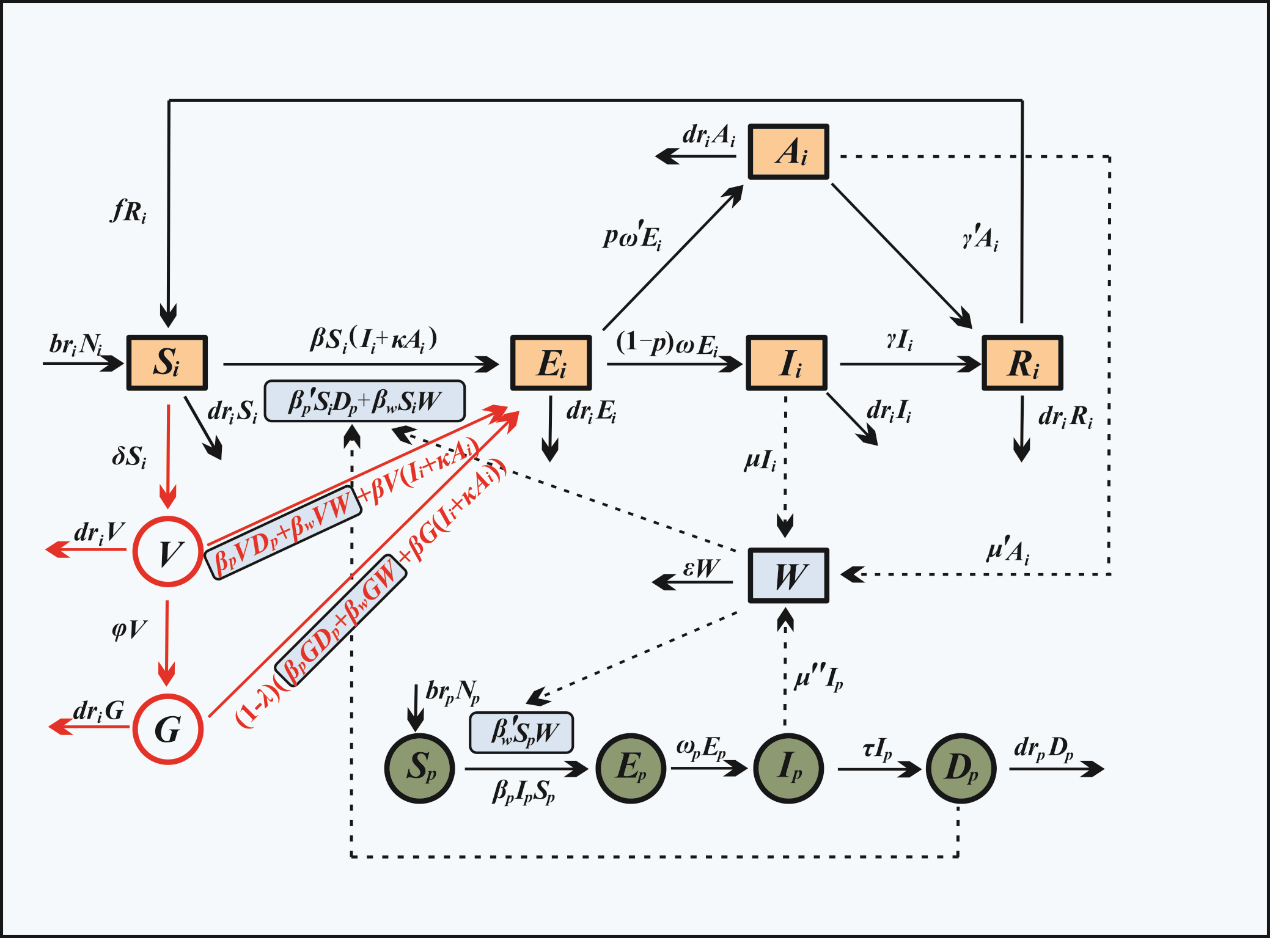


**Fig. S1** Establishing the transmission dynamics of the vaccination intervention model of hepatitis E.


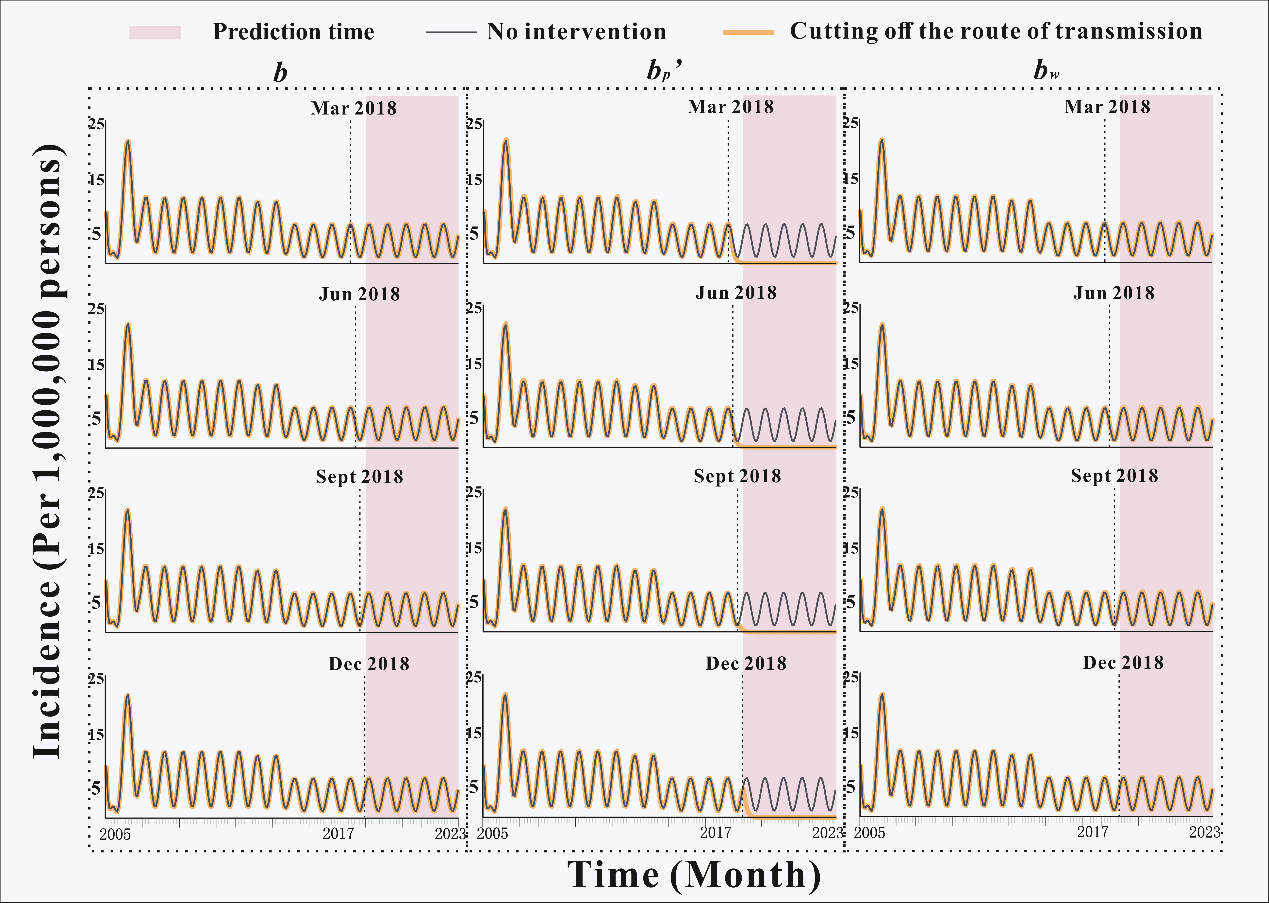


**Fig. S2** Effects of cutting the transmission routes on the incidence of hepatitis E of Zhenjiang City.


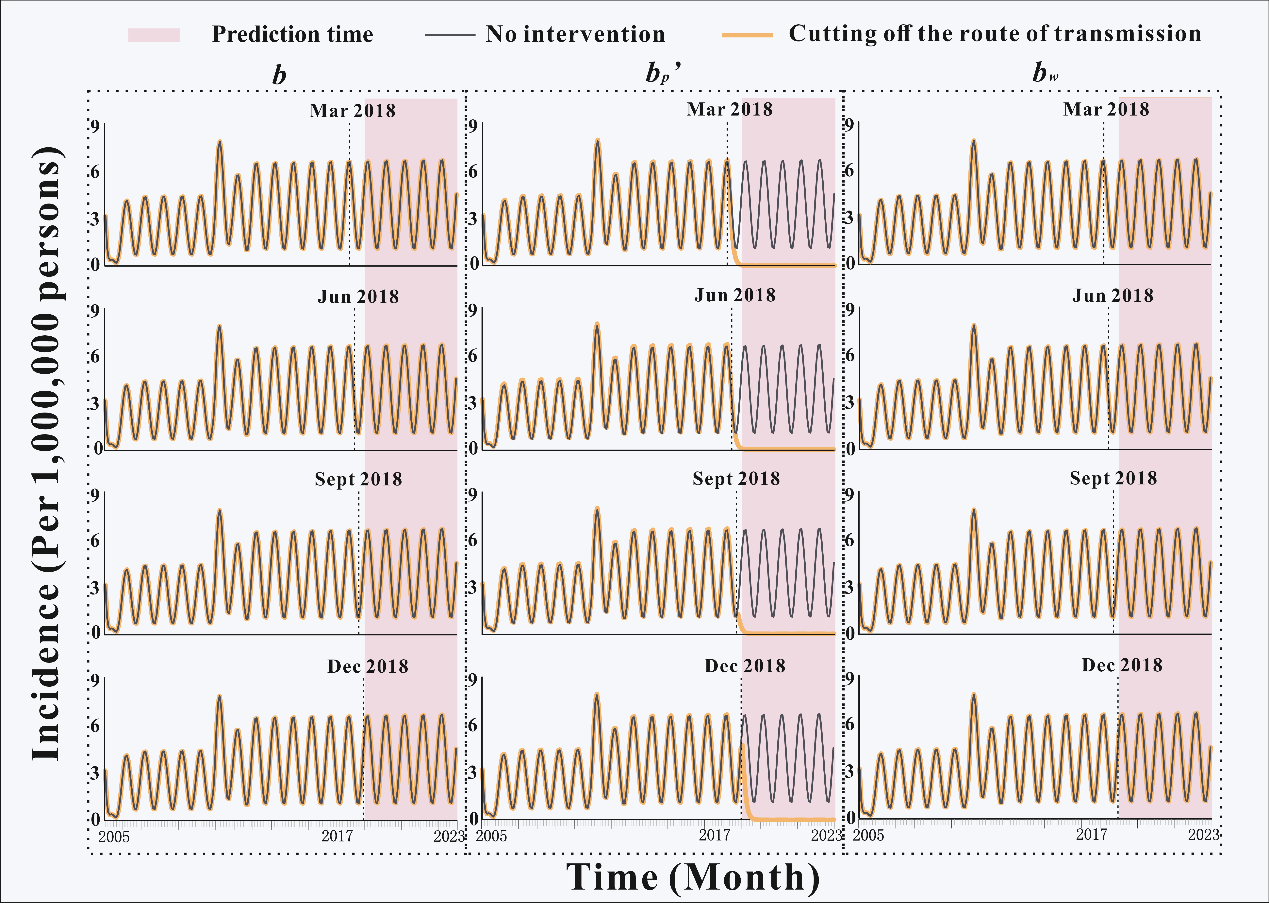


**Fig. S3** Effects of cutting the transmission routes on the incidence of hepatitis E of Yancheng City.


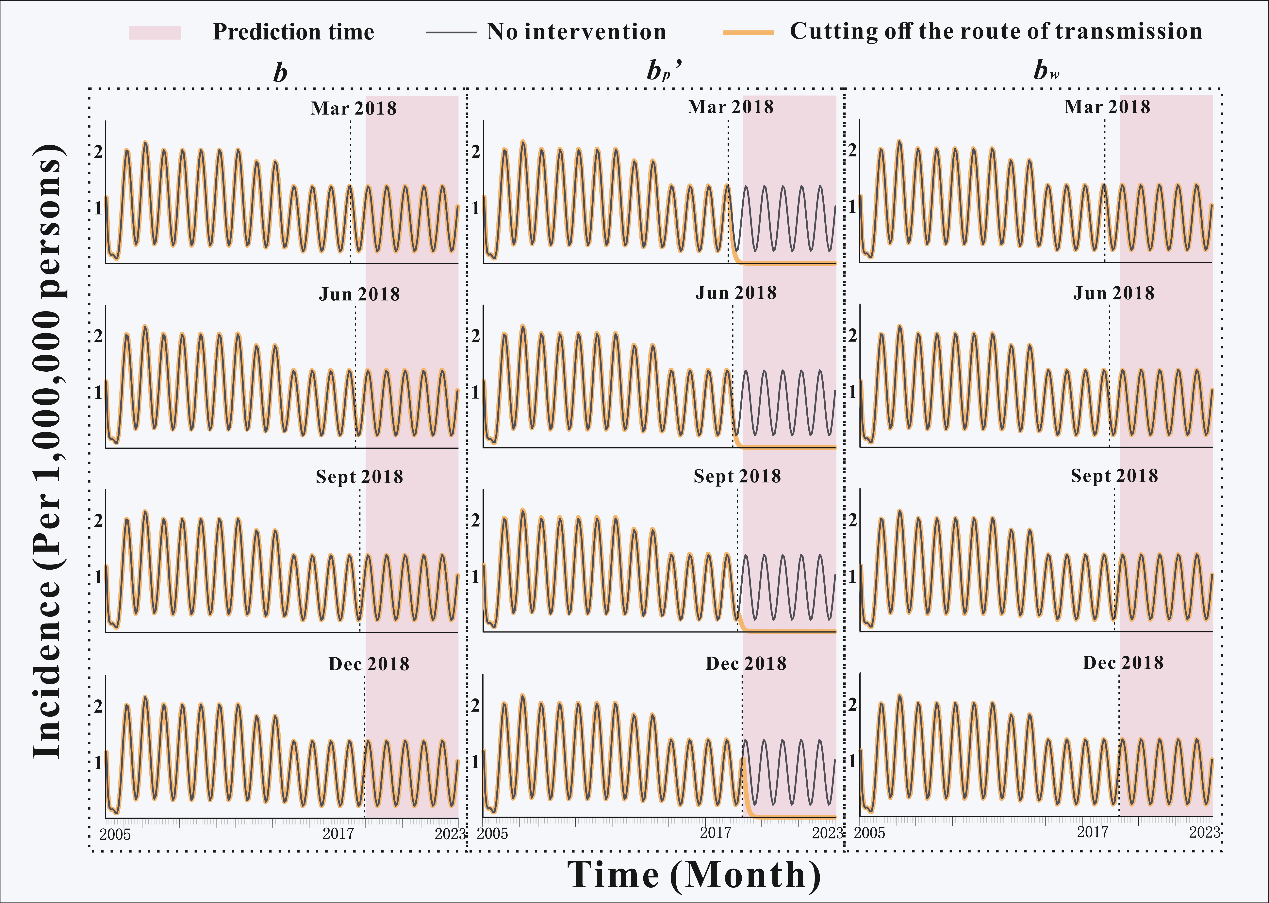


**Fig. S4** Effects of cutting the transmission routes on the incidence of hepatitis E of Wuxi City.


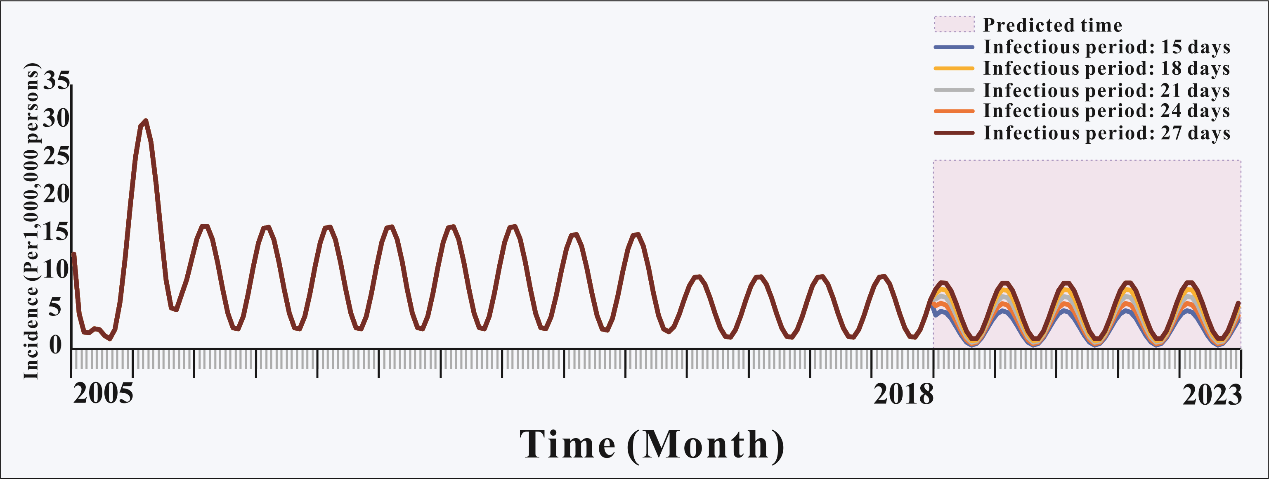


**Fig. S5** Simulation of shortening the incidence of hepatitis E infection of Zhenjiang City.


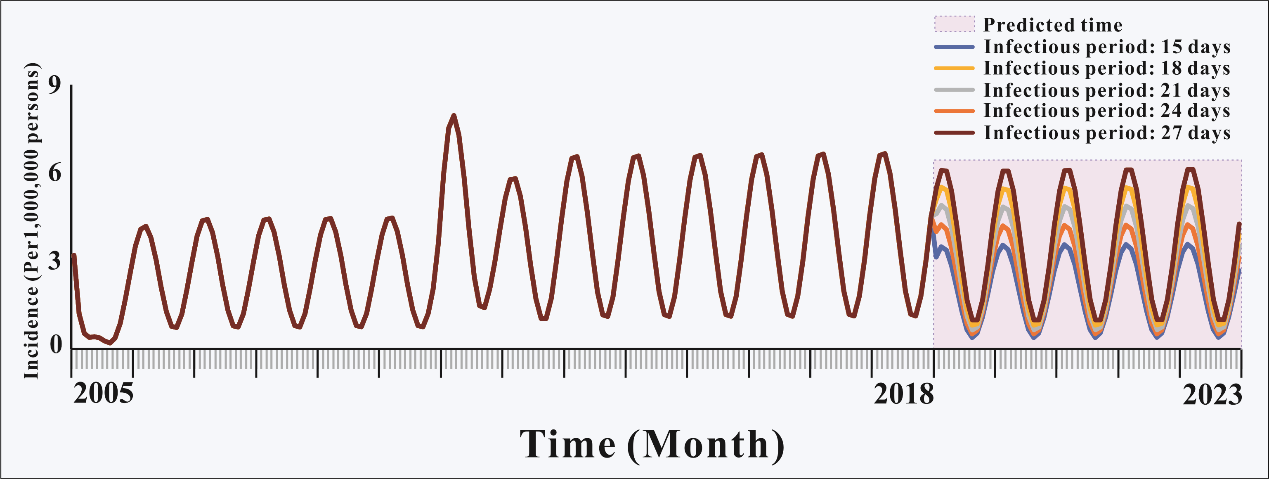


**Fig. S6** Simulation of shortening the incidence of hepatitis E infection of Yancheng City.


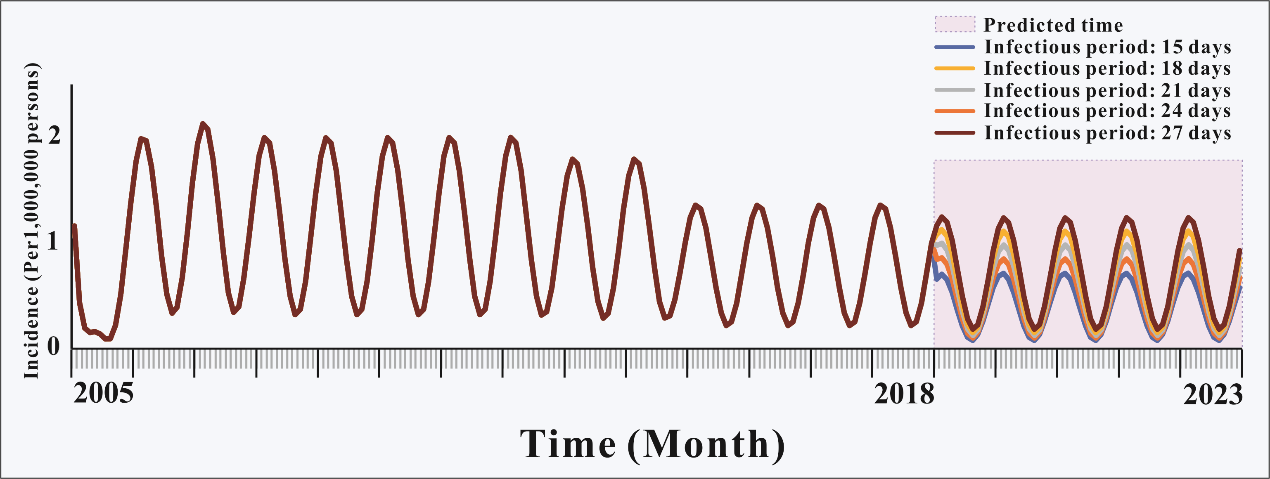


**Fig. S7** Simulation of shortening the incidence of hepatitis E infection of Wuxi City.


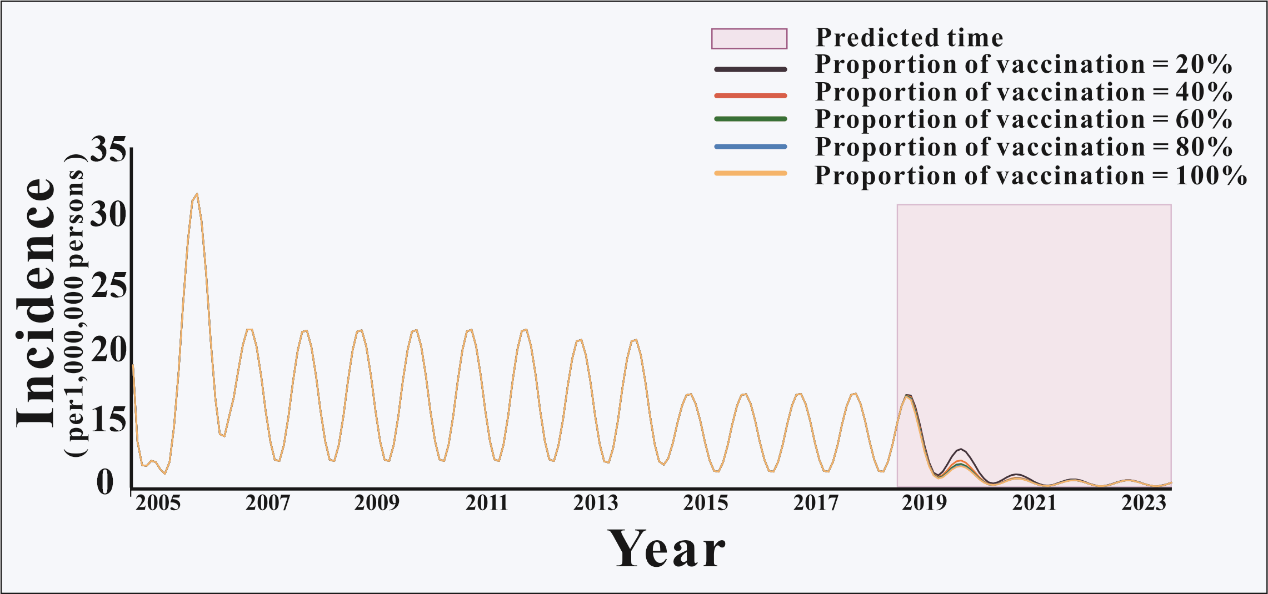


**Fig. S8** Effects of different vaccination coefficients on the incidence of hepatitis E infection of Zhenjiang City.


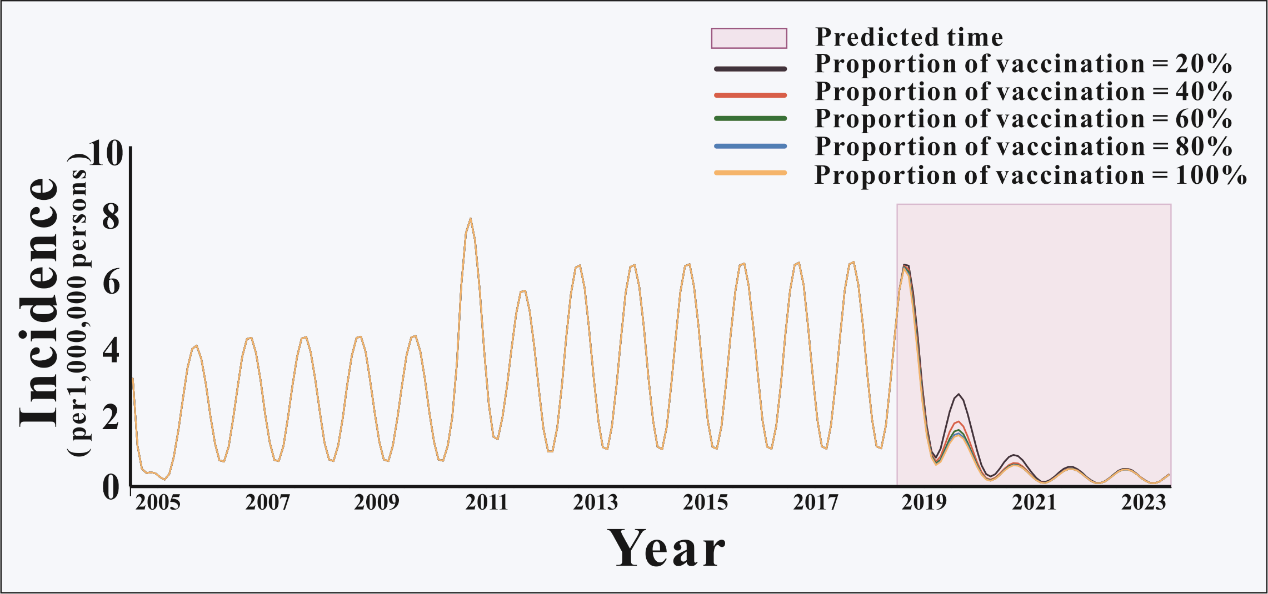


**Fig. S9** Effects of different vaccination coefficients on the incidence of hepatitis E infection of Yancheng City.


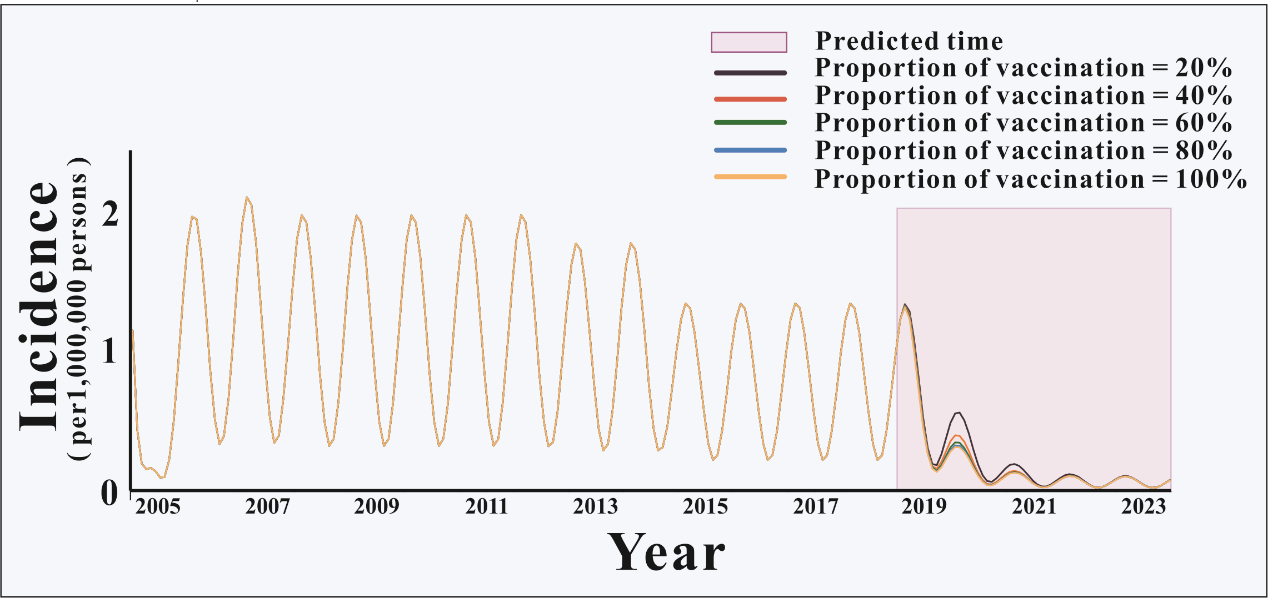


**Fig. S10** Effects of different vaccination coefficients on the incidence of hepatitis E infection of Wuxi City.


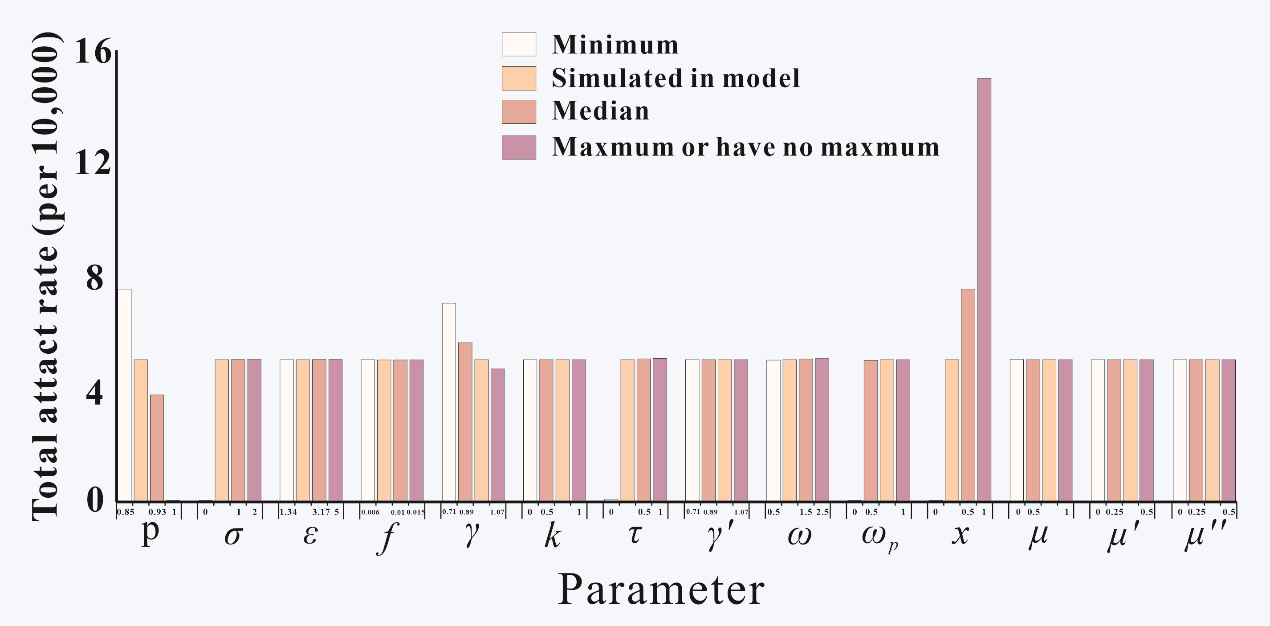


**Fig. S11** The sensitivity analysis of parameter.
